# Status of Antibiotic Susceptibility of Polymyxin B And Colistin in the Treatment of Carbapenem Resistant Gram Negative Bacterial Infections: An Observational Study

**DOI:** 10.31729/jnma.v63i2091.9226

**Published:** 2025-11-30

**Authors:** Ashish Kumar Bhattarai, Khilasa Pokharel, Marina Vaidya Shrestha

**Affiliations:** 1 Department of Pharmacology, Kathmandu Medical College and Hospital, Kathmandu, Nepal; 2 Department of Microbiology, Kathmandu Medical College and Hospital, Kathmandu, Nepal; 3 Department of Community Medicine, Kathmandu Medical College and Hospital, Kathmandu, Nepal

**Keywords:** *antibiotic sensitivity*, *carbapenem resistance*, *colistin*, *gram negative bacteria*, *multidrug resistance*, *polymyxin b*

## Abstract

**Introduction:**

The emergence and rapid spread of multidrug-resistant gram-negative bacteria is a global concern. Polymyxin B and Colistin have gained significance due to the scarcity of new antibiotics and the high morbidity and mortality associated with carbapenem-resistant gram-negative bacteria. This study aims to assess the antibiotic susceptibility of Colistin and Polymyxin B against carbapenem-resistant gram-negative bacteria.

**Methods:**

This was a descriptive cross-sectional study conducted in a tertiary center of Nepal, from June to August 2025. Specimen resistant to any of the carbapenem drugs at the Clinical Laboratory was evaluated. The incidence of susceptibility or resistance of the Polymyxin and Colistin in the carbapenem-resistant gram-negative bacteria was recorded and assessed.

**Results:**

A total of 1,412 Gram-negative bacterial samples were analyzed within the predetermined study period. Among these samples, the prevalence of 395 (27.97%) was from carbapenem-resistant Gram-negative bacteria. There were 185 (46.84%) carbapenem-resistant *Klebsiella* species, followed by 105 (26.60%) *Acinetobacter* species. Both Polymyxin B and Colistin showed 100% susceptibility across all carbapenem-resistant gram-negative bacteria species and sample sources evaluated.

**Conclusions:**

Polymyxin B and Colistin continue to be vital last-line treatments for carbapenem-resistant gram-negative bacteria. This study found a susceptibility rate of 100% to colistin and polymyxin B among carbapenem-resistant gramnegative bacteria. To maintain the efficacy and reliability of these antibiotics, it is essential to use them judiciously, conduct routine susceptibility testing, and implement ongoing surveillance.

## INTRODUCTION

The rising prevalence of antibiotic resistance is a global issue affecting both human and animal health. Misuse of antimicrobials directly contributes to the increase in antimicrobial resistance.^1^

Infections from multidrug-resistant (MDR) and extensively drug-resistant (XDR) Gram-negative bacteria, such as carbapenem-resistant *Enterobacterales, Pseudomonas aeruginosa,* and *Acinetobacter baumannii,* have become a significant public health challenge.^2^ Polymyxin B sulfate and colistin were rarely used for many years due to the availability of less toxic antibiotics. They are now recommended only for serious systemic infections caused by Gram-negative bacteria resistant to other treatments.^3^ The main toxic effects that limited the usage of polymyxins were nephrotoxicity and neurotoxicity.^4^

Polymyxin B and Colistin sulfate are both efficacious in the treatment of Carbapenem-resistant gram-negative bacteria (CR-GNB).^5^ This study aimed to evaluate the antibiotic susceptibility of Colistin and Polymyxin B for treating CR-GNB at a tertiary care hospital in Nepal.

## METHODS

This was a descriptive cross-sectional study conducted in the Kathmandu Medical College Teaching Hospital (KMCTH) from June to August 2025. The study was conducted obtaining the data from the records from the Clinical Microbiology laboratory of KMCTH. The samples were sent from the wards, intensive care unit (ICU) of different departments to the laboratory. All the Clinical specimens of the carbapenem-resistant Gram-negative bacteria presented to the Clinical Microbiology Laboratory of KMCTH was evaluated during that study period. This study was approved by the Institutional Review Committee of the Kathmandu Medical College (Ref: 25052025/14). The permission from the clinical microbiology department and hospital Administration was received. Only the relevant information was obtained from the laboratory reports. And, no information was taken and published which could break anonymity of patients. Incomplete records, and patients who have received polymyxin B and Colistin in the last 30 days were excluded.

Data was collected from electronic and manual patient registration. The following variables were recorded: Age, gender, site of isolation of the organism, specimen, isolated organism and concomitant antibiotic therapy was obtained. All the carbapenem-resistance organisms (CRO) samples reported from culture in Microbiology Laboratory were re-processed to check its antibiotic susceptibility for Polymyxin B and Colistin.

The CRO isolates were identified by guidelines recommended by Centers for Disease Control and Prevention (CDC), which define CRO as multidrug-resistance organisms (MDRO) that are resistant to at least one of the carbapenem antibiotics (ertapenem, meropenem, doripenem or imipenem) or produce a carbapenemase.^6^

Antibiotic sensitivity testing was done with the Use of Mueller-Hinton agar for susceptibility testing of Polymyxin B and Colistin. Antibiotic disks were applied according to standardized guidelines.

Census sampling was used. All the specimen presented to the laboratory during that period was recorded and assessed. Data were collected weekly from the laboratories, focusing on resistant cases of gram-negative bacteria. Categorical encoding of the data were done after validation. Checking and removal of duplicate entries were performed. Data entry was performed in Excel, and analysis was conducted using the Statistical Package for the Social Sciences (SPSS version 25). Descriptive statistics like mean, median, and percentage were used to summarize the data. Bar graphs and pie charts were used to visually represent the distribution of sensitivity and resistance rates among different bacterial species.

## RESULTS

**Figure 1:**
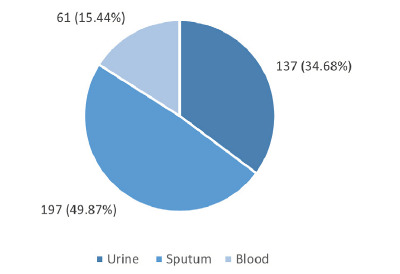
Specimen-Wise Distribution of Carbapenem-Resistant Gram-Negative Bacteria (n=395).

A total of 1,412 Gram-negative bacteria samples were analyzed within the predetermined study period. Among these samples, the prevalence of 395 (27.97%) were from carbapenem-resistant Gram-negative bacteria. The mean age of the patients was 45.57±24.26 years and the median age was 48.00. The reports from male were 252 (63.80%) whereas that from female were 143 (36.20%). Samples were collected from patients ranging in age from one day old to 92 years old. There were 197 (49.87%) samples from sputum (Figure 1).

**Figure 2:**
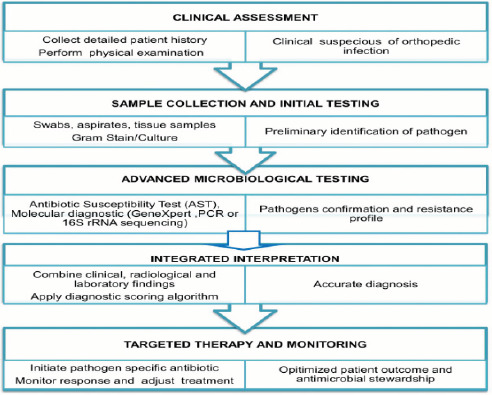
Distribution of Different Types of Isolated Organisms (n=395).

The Gram-negative bacteria showing resistance to Carbapenem were *Klebsiella* species, *Acinetobacter* species, *E. coli, Pseudomonas aeruginosa*, and *Enterobacter* species. There were 185 (46.84%) *Klebsiella* species, followed by 105 (26.58%) *Acinetobacter* species, resistant to carbapenem (Figure 2).

**Table 1:** Carbapenem resistant isolated organisms and its sample categories (n=395).

	Samples		
Isolated organisms	Urine	Sputum	Blood
*Klebsiella species*	67(48.90%)	75(38.07%)	43(70.49%)
*E. Coli*	49(35.76%)	16(8.12%)	1(1.63%)
*Pseudomonas aeruginosa*	14(10.21%)	20(10.15%)	1(1.63%)
*Acinetobacter species*	5(3.64%)	85(43.14%)	15(24.59%)
*Enterobacter species*	2(1.45%)	1(0.50%)	1(1.63%)

**Table 2:** Isolated organisms and their sensitivity with Polymyxin B and Colistin (n=395).

Isolated organisms	Sensitivity of Polymyxin B and Colistin
*Klebsiella species*	185(100%)
*Acinetobacter species*	105(100%)
*E coli*	66(100%)
*Pseudomonas aeruginosa*	35(100%)
*Enterobacter species*	4(100%)

In urine samples, 67 (48.90%) *Klebsiella* species were isolated, 49 (35.76%) *E. coli* were isolated, followed by 14 (10.21%) *Pseudomonas aeruginosa.* In sputum samples, 85 (43.14%) *Acinetobacter* species were isolated, followed by 75 (38.07%) *Klebsiella* species and 20 (10.15%) *Pseudomonas aeruginosa.* In blood samples, 43 (70.49%) *Klebsiella* species were obtained, followed by 15 (24.59%) *Acinetobacter* species. Each of *E. coli, Enterobacter* species, and *Pseudomonas aeruginosa* contributed 1 (1.6%) isolate from blood (Table 1).

Isolated of various species obtained from urine, sputum and blood samples were tested for antibiotic sensitivity of Polymyxin B and Colistin. There were 185 (100%) *Klebsiella* species, 105 (100%) *Acinetobacter* species and 66 (100%) *E coli,* sensitive to Polymyxin B and Colistin (Table 2).

## DISCUSSION

Carbapenems are class of Beta lactam antibiotics that demonstrate broader antimicrobial spectrum than penicillins, cephalosporins, and P-lactamase inhibitor combinations. Imipenem, panipenem, and doripenem are potent antibiotics against Gram-positive bacteria whereas meropenem, biapenem, and ertapenem, are slightly more effective against Gramnegative organisms.^7^ Meropenem is a type of Carbapenem group of antibiotics registered in Nepal and also included in national list of essential medicines (NLEM) 2021 revision. It is included as reserve group of antibiotics along with Polymyxin B, whereas colistin is kept in the complimentary list.^8^ Meropenem effectively treats different Gram-negative bacilli, such as *K. pneumoniae, Enterobacter* spp., and *P. aeruginosa*. Meropenem alone and with combination with other agents showed higher cure rates and had significantly lowered the patient mortality.^9^ Meropenem and imipenem were the most commonly used Carbapenems in this hospital and were therefore considered representative of carbapenem use in this study.

The spectrum of activity are similar for all Carbapenems, with little variances in activities of individual agents except for ertapenem. The absence or reduced expression of two major porins in combination with various beta-lactamases and alteration of some penicillin binding proteins (PBPs) have been associated in carbapenem resistance.^10^

Polymyxin B and Colistin both are structurally similar polymyxins. Both of them have re-emerged in clinical practice to treat infections caused by multi-drug (MDR) or extensively-drug-resistant (XDR) Gram-negative bacteria (GNB). However, since Colistin is administered as a prodrug, there are major pharmacokinetic differences between both polymyxins that may potentially determine different clinical and microbiological outcomes.^11^ Polymyxin B has the highest sensitivity, and the combination regimen based on Polymyxin B is the preferred treatment for systemic or local infections caused by the *Klebsiella pneumonia, Pseudomonas aeruginosa, Escherichia coli* and *Acinetobacter baumannii.^12^* But, with the excessive use of polymyxin B, the Klebsiella pneumoniae Carbapenemase (KPC) producing *Klebsiella pneumoniae* resistant to polymyxin B has also been reported worldwide.^13^ Eschenchia *coli* also has resistance to polymyxin B.^14^

CR-GNB possess a high resistance rate against a wide range of antibiotics, further limiting the antibiotic options available for patients. CRE (carbapenem-resistant *Enterobacteriaceae),* CRAB (carbapenem-resistant *Acinetobacter baumannii),* and CRPA (carbapenem-resistant *Pseudomonas aeruginosa)* are classified as pathogens posing a significant threat to human health.^15^

Treatment for CR-GNB infections with Polymyxin B, either alone or in combination with an optimal dose, should be initiated promptly to enhance clinical efficacy and minimize the risk of drug resistance. Guidelines for sepsis, emphasize that once the diagnosis and etiological examination are confirmed, antimicrobial treatment should commence immediately. Additionally, the antibiotic regimen should be adjusted early based on bacterial susceptibility results. Delayed treatment, even with appropriate drug selection, can lead to increased mortality and extended hospitalization.^16^ In this study, the sensitivity was found to be 100%, which supports the recommendation for physicians to initiate Polymyxin B as early as possible as empirical therapy against CR-GNB, even before receiving culture and sensitivity reports.

In this study, the mean age calculated from the collected patient reports was 45.57 ± 24.26. The reports from one day older child to 92 years old was obtained. This indicated the sternness of the problem, even a child is infected with MDR in their born day as also described in different systematic reviews and meta-analysis.^17^ Most of the samples were obtained sputum (49.90%) followed by urine (34.70%). This indicates most of the CR-GNB were from the respiratory tract infections.

The prevalence of the most CR-GNB were that of *Klebsiella* species 185 (46.80%) followed by *Acinetobacter* species 105 (26.60%). Other CR-GNB showing resistance was from *E.coli* 66 (16.70%), *Pseudomonas* species 35 (8.90%) and *Enterobacter* species 4 (1.00%). A study was done by Siwakoti S. et al. in one of the tertiary center of Nepal. The incidence rate of multi-drug resistant gram-negative bacteria infections was 47% among admitted patients. *Acinetobacter species* (41%) was the commonest followed by *Klebsiella species* (28%) and *Pseudomonas aeruginosa* (21%). This study also shows that there was very high incidence of multi-drug resistant gram-negative bacterial infections was remarkably high in that intensive care unit. ^18^

Out of 1412 samples of Gram-negative bacteria in different specimens, 395 (27.97%) were identified as CR-GNB in this study. Similar finding was obtained with the study done by Konwar D in one of study done in the tertiary care center in Nepal. The findings were the prevalence of CR-GNB was found to be (26.26%). In contrast the highest occurrence of the isolated bacteria were of *Acinetobacter* species (44.57%) followed by *Klebsiella* species (25.29%) in their study.^19^

In the study conducted by PM Emeka using the Broth Microdilution (BMD) assay, a similar resistance pattern was observed between Colistin and Polymyxin B. This was linked to the presence of chromosomal mcr-1 genes in the study region.^20^ As an effect, susceptibility testing for Polymyxin B and Colistin in CR-GNB is performed together to assess both susceptibility and resistance simultaneously.

The occurrence of carbapenem resistant *Klebsiella* species were present at the highest number in the urine 67 (48.90%) and blood samples 43 (70.49%). Highest number percentage of organism obtained from sputum was from *Acinetobacter* 85 (43.14%). *E.coli, Pseudomonas* and *Enterobacter* had negligible occurrence in the blood. It means *Klebsiella* resistant to Carbapenem was isolated from respiratory diseases while *Acinetobacter* was isolated from urinary tract infections in the highest number. This study demonstrated a 100% sensitivity of Polymyxin B and Colistin across all species of CR-GNB and from all recorded sample sources. Consequently, no further comparison or evaluation among these drugs was necessary. The results indicate that, to date, the antibiotic sensitivity of these two agents in this tertiary hospital remains satisfactory. However, because these drugs are last-resort options, routine surveillance of their efficacy and resistance is essential. As mentioned, numerous studies worldwide have highlighted the emergence of resistance to these drugs and methods to combat them.

One study done in south Asian population suggests that combining intravenous and nebulized polymyxin B significantly improved microbial clearance, reduced ICU length of stay, and decreased mortality in critically ill South Asian patients with MDR *K. pneumoniae* related ventilator associated pneumonia. These results suggest that the dual route regimen is an effective management strategy for this infection.^21^

Regular periodic surveillance of antibiotic susceptibility patterns for Polymyxin B and Colistin among carbapenem-resistant Gram-negative bacteria is required to monitor trends and make treatment protocols. Establish or strengthen antibiotic stewardship programs to promote the judicious use of Polymyxin B and Colistin, minimizing the risk of resistance development. Encourage research into combination therapies that incorporate Polymyxin B and Colistin with other antibiotics to enhance efficacy and reduce the potential for resistance.

Conducting the study at a single tertiary care hospital may limit case diversity and reduce the applicability of the findings to other healthcare settings. Additionally, the data collection period might not capture seasonal variations or changes in antibiotic resistance patterns over time. The study also may not delve deeply into the underlying mechanisms of resistance, which restricts insights into the reasons for certain bacteria being resistant. Furthermore, by focusing solely on antibiotic sensitivity without correlating it to clinical outcomes, the study may not offer a comprehensive understanding of treatment effectiveness.

## CONCLUSION

Polymyxin B and colistin are vital salvage options for treating CR-GNB infections. In this study, the drug susceptibility rate was 100%. To maintain the efficacy and reliability of these agents, sensible and rational use, routine susceptibility testing, and ongoing surveillance are essential. Additionally, there is a pressing need for research and development of new antibiotics to prepare for future challenges posed by antimicrobial resistance to these last-resort treatments.

## Data Availability

The data are available from the corresponding author upon reasonable request.
